# Stoichiometric niche, nutrient partitioning and resource allocation in a solitary bee are sex-specific and phosphorous is allocated mainly to the cocoon

**DOI:** 10.1038/s41598-020-79647-7

**Published:** 2021-01-12

**Authors:** Michał Filipiak, Michal Woyciechowski, Marcin Czarnoleski

**Affiliations:** Faculty of Biology, Institute of Environmental Sciences, Jagiellonian University, Kraków, Poland

**Keywords:** Element cycles, Food webs, Ecology, Biodiversity, Biogeochemistry, Community ecology, Conservation biology, Ecophysiology, Ecosystem ecology

## Abstract

Life histories of species may be shaped by nutritional limitations posed on populations. Yet, populations contain individuals that differ according to sex and life stage, each of which having different nutritional demands and experiencing specific limitations. We studied patterns of resource assimilation, allocation and excretion during the growth of the solitary bee *Osmia bicornis* (two sexes) under natural conditions. Adopting an ecological perspective, we assert that organisms ingest mutable organic molecules that are transformed during physiological processes and that the immutable atoms of the chemical elements composing these molecules may be allocated to specific functions, thereby influencing organismal fitness and life history. Therefore, using the framework of ecological stoichiometry, we investigated the multielemental (C, N, S, P, K, Na, Ca, Mg, Fe, Zn, Mn, Cu) compositions of six components of the bee elemental budget: food (pollen), eggs, pupae, adults, cocoons and excreta. The sexes differed fundamentally in the assimilation and allocation of acquired atoms, elemental phenotypes, and stoichiometric niches for all six components. Phosphorus, which supports larval growth, was allocated mainly (55–75%) to the cocoon after larval development was complete. Additionally, the majority (60–99%) of the Mn, Ca, Mg and Zn acquired during larval development was allocated to the cocoon, probably influencing bee fitness by conferring protection. We conclude that for holometabolous insects, considering only the chemical composition of the adult body within the context of nutritional ecology does not provide a complete picture. Low ratios of C to other nutrients, low N:P and high Na concentrations in excreta and cocoons may be important for local-scale nutrient cycling. Limited access to specific nutritional elements may hinder bee development in a sex-dependent manner, and N and P limitations, commonly considered elsewhere, may not play important roles in *O. bicornis*. Sexual dimorphism in nutritional limitations due to nutrient scarcity during the larval stage may influence bee population function and should be considered in bee conservation efforts.

## Introduction

The enormous diversity of life cannot be fully understood without considering the selection imposed by the capacity to acquire adequate types and amounts of resources, specifically, energy and matter, for the production of new tissue (in individuals directly or in offspring) and the maintenance of existing tissue^1–3^. Previous studies have focused on the varying supply of and demand for energy across environments, populations and species and on the potential fitness consequences of energy allocation to vital organismal functions^1,4,5^. Here, we argue that this energy-oriented view of life is incomplete without the consideration of other factors that affect physiology and survival. An organism searching for an energy supply needs to also acquire a body-building supply, which can be challenging and necessitate a trade off regarding access to these two types of supply^2^. The challenges appear even more complex if we consider that the relative importance of each type of supply can shift throughout life; e.g., compared to energetically limited adults, growing juveniles can experience severe limitations imposed by limited access to body-building matter^6,7^.

Supply is available in the environment in the form of foods composed of organic molecules. These molecules are short lived in food webs because they are mutable; when digested, they undergo complex biochemical reactions inside the organism^2,8^. Every molecule is composed of a specific set of atoms of chemical elements^2^. These immutable atoms cycle in food webs in endless loops, being ceaselessly incorporated into molecules that are being ceaselessly degraded; this process is called the biogeochemical cycle^9–11^. When studying the life histories of organisms, we can use the proportions of various atoms as the metrics to investigate questions about organismal strategies related to nutrient assimilation, allocation and excretion^2^. In this work, we adopt this approach and the biochemistry-oriented view that digested molecules are processed and that the immutable atoms they comprise are allocated to specific functions. This approach makes our predictions ecologically and evolutionary relevant.

The stoichiometric approach to organisms and their life cycles considers that organismal functions involve an array of biochemical reactions that are subject to limitation unless the atomic composition of products is balanced by the atomic composition of reactants^2^. Ratios of different elements in a body, also called the elemental phenotype^12^, differ among organisms (e.g., sexes, species), reflecting differences in body structure and function^13–15^. We can predict that a match between the elemental ratios of an organism’s body and its food has a fundamental impact on fitness-related organismal functions^15–17^. Importantly, this match could have especially pronounced fitness consequences if the nutritional quality of resources obtained by an organism at the juvenile stage strongly determines its future life history characteristics during the adult stage^6,7^. Even more important, this dependency is a common rule that applies to all eumetazoans (invertebrates and vertebrates)^2^. Consequently, we expect that when searching for the stoichiometrically balanced diet, organisms with specific elemental phenotypes would prefer specific food sources, taking adequate behavioral actions to reach these sources. At a larger scale, the match between stoichiometric characteristics of food and consumers’ bodies would shape ecological interactions and ecosystem functioning^2,17^.

To a large extent, our current understanding of ecological stoichiometry is based on research that has considered between-species differences (e.g.,^18,19^) and aquatic ecosystems (e.g.,^18,20,21^). Therefore, shifting the focus toward terrestrial systems and within-species variance would provide a fuller picture of the effects of the stoichiometric match between resource demand and supply. Emerging evidence suggests that much of the within-species differences in the chemical composition of the body is attributed to sexual dimorphism^22–24^. Indeed, processes involved in life-history evolution and population dynamics may be sex-specific, leading to different nutritional limitations for each sex^17,22^.

**Figure 1:**
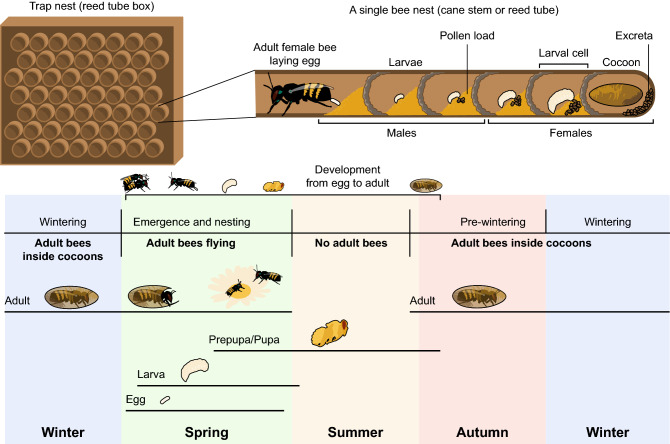
Nesting biology of solitary *Osmia* bees (applicable to various species inhabiting different continents).

Addressing these effects, we studied the assimilation, excretion, and allocation of C, N, S, P, K, Na, Ca, Mg, Fe, Zn, Mn, and Cu during development in males and females of the red mason bee, *Osmia bicornis* L. This bee is a generalist solitary wild bee inhabiting almost the whole of Europe, the northern part of Africa and western Asia. Females of *O. bicornis* establish their nests in narrow spaces, such as the hollow stems of plants (Fig. 1; see supplement for details). Each nest consists of a set of incubation chambers (larval cells), with each chamber containing one egg and pollen deposit; larval cells located at the back side of the nest are filled with female eggs, and larval cells located at the front side of the nest are filled with male eggs. The biology of mason bees enabled us to perform our study under natural conditions, collecting the entire amount of food eaten by larvae (this alone is unprecedented) and feces excreted by larvae during development, as well as eggs, pupae, adult bees, and cocoons. We measured the concentration and mass of the studied elements in each component of the elemental budget (eggs, pollen, pupae, adults, excreta, cocoons), which was used to evaluate the assimilation of elements from pollen eaten by larvae, excretion and the further transfer of elements to an adult body and cocoon, addressing potential sex differences in these processes. We stress here that information on the naturally occurring assimilation rates of elements is virtually nonexistent in the literature but is essential for further development of the ecological stoichiometry framework^25–27^. Importantly, mason bees spend a considerable part of their life cycle inside cocoons (Fig. 1), suggesting that the chemical composition of their cocoons is under strong selective control. Indeed, it was suggested that cocoons are an important sink for the noncarbon element pool assimilated and used during larval development^28^. Therefore, we used our data to explore how different elements obtained by larvae from pollen are further allocated to the adult body vs. cocoons, considering that this allocation can be sex-specific. This helped us to identify potential allocation decisions and trade-offs involved in elemental transfer and to determine whether these phenomena occur similarly in male and female bees. It should be emphasized that to date, the majority of research on bees has focused on the nutritional needs of adults^6,23^. Therefore, our study of the elemental budget of developing bees has important implications for a better understanding of plant–bee interactions.

## Results

Note that when reporting statistical results here, we also refer to the supplement whenever we want to draw attention to detailed information on the actual concentrations of elements and atomic ratios *C:N*, *C:P*, *N:P*.

### Elemental budget

**Figure 2:**
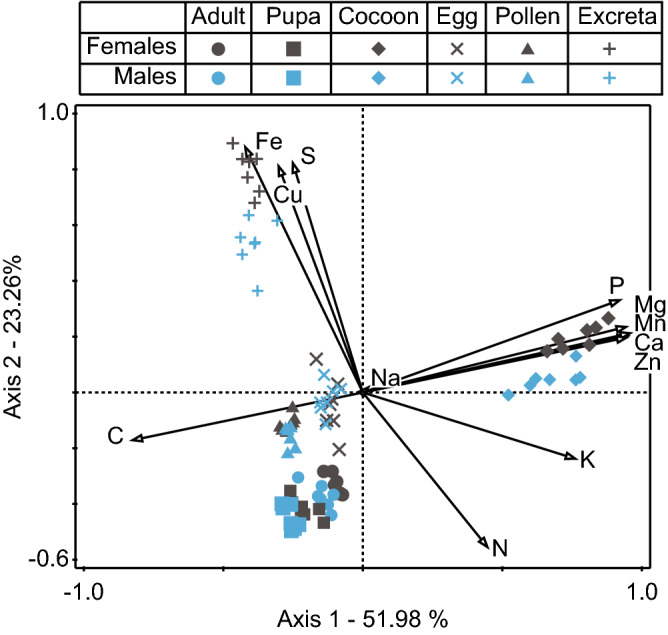
General pattern of elemental partitioning between different components of the elemental budget. PCA plot—the whole elemental budget of *O. bicornis* and stoichiometric relations between its components; calculated according to the concentrations of elements. Note that the body and cocoon add up to the total production that is built based on the food eaten during larval growth—pollen. The first two axes are presented. The percentage of variation explained is given for both axes. The lengths of vectors represent the contribution of every element to the pattern shown (longer vector = greater contribution). The direction of the vector shows the axis to which it contributes. Detailed results are shown in the supplement. The adult body is the component richest in N, cocoon is the component richest in P, Mg, Mn, Ca and Zn, and excreta are the richest in Fe, S and Cu. *C:P* ratio is decreasing from left to right along the wide line between pupa and cocoon. A detailed analysis of sexual differences is shown in Fig. 3.

In the first step, we obtained the overall picture of the elemental budget by analyzing all the data (concentrations of elements) together with a PERMANOVA followed by a PCA. The results of the PERMANOVA (Table 1) showed that sexes had different stoichiometric characteristics and that the nature of these sex differences was dependent on the analyzed budget component (indicated by the significant interactions between sex and the budget components). The results of the PCA (Fig. 2) showed that the variance was primarily driven by differences among budget components, with sex differences playing a small role. Therefore, we used these results to further investigate the stoichiometric differences between budget components while exploring sex differences in detail with another set of analyses (see next paragraph). Given that pollen and eggs are the only source of matter for developing bees, we analyzed the results shown in Fig. 2 focusing on stoichiometric dissimilarities between pollen and eggs in relation to the remaining budget components. Generally, Fig. 2 shows that pollen and eggs clustered close to pupae and adults forming one cluster composed of these four separate groups, being more similar to each other in terms of multi-elemental stoichiometry than to other components of the elemental budget. Among these four components, pupae and adults were exceptionally rich in N (pupae: approximately 2.5- to 1.5-fold and adult: approximately 3.5- to 2-fold higher concentration than pollen–egg, respectively, see supplement), while pupae and excreta were exceptionally rich in C (pupae had also the highest *C:P* ratio; see supplement). Note that the pupa-adult difference must indicate losses of C through respiration during metamorphosis. Compared to these four budget components taken together, cocoons were exceptionally rich in P (approximately seven to fivefold higher concentration than in pollen–egg, respectively; see supplement), Mg (13- to 10-fold), Mn (14- to 36-fold), Ca (13- to 4-fold), Zn (7.5- to 2.5-fold), K (3.5- to 1.6-fold) but poor in C, while excreta were exceptionally rich in Fe, Cu and S compared to all other budget components (Fe approximately four to fivefold higher concentration than in pollen|egg, respectively, Cu 3- to 2.5-fold and S 3- to 1.5-fold; see supplement). Figure 2 shows that Na did not contribute significantly to either axis 1 or 2. Nevertheless, the concentration of Na was an order of magnitude higher in eggs than in other budget components (see supplement).

**Table 1:** In the red mason bee *Osmia bicornis*, the stoichiometry of 12 elements differed between components of the elemental budget (pollen loads, excreta, eggs, pupae, adults, cocoons), but the nature of these differences further was dependent on sex (significant A × B interaction).

	df	F	P
Component of elemental budget (A)	5	288.7	0.0001
Sex (B)	1	13.356	0.0001
A × B interaction	5	7.83	0.0001
Residual	72		

The results of two-way PERMANOVA.

In the next step, we focused primarily on sex differences, analyzing data for each component independently with the help of a separate PCA, followed by a t-test on the calculated axis scores. The patterns shown in the graphical representation of PCAs were confirmed by t-tests computed independently for the 1st- and 2nd-axis scores (p < 0.05). The results of these analyses are shown below.

#### Eggs

**Figure 3:**
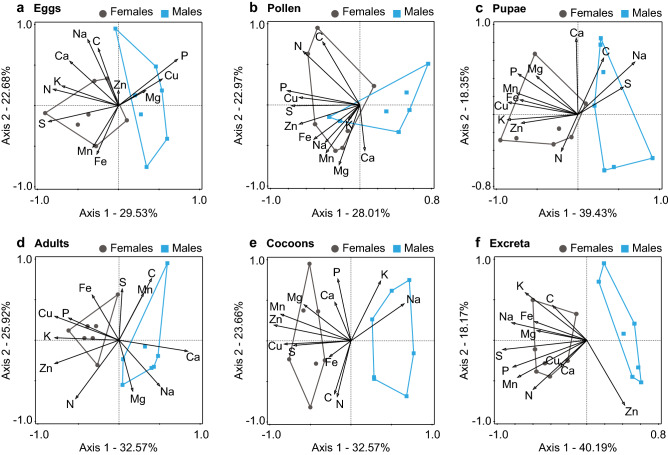
The sexes differ in the stoichiometry of every component of their elemental budget. PCA plots—multivariate analysis of the stoichiometric relations of the *O. bicornis* elemental budget; calculated according to the concentrations of elements. The first two axes are presented. The percentage of variation explained is given for both axes. The patterns shown in the graphical representation of PCAs were confirmed by t-tests computed independently for the 1st- and 2nd-axis scores (p < 0.05). Detailed results are shown in the supplement.

The first two principal axes of the PCA explained 52.21% of the variation (Fig. 3a). Eggs formed two groups along the 1st axis, explaining 29.53% of the variation. The elements contributing mostly to this shift were S (1st-axis loading: 0.86), N (0.80), and K (0.70), with higher concentrations in females, and P (0.73) and Cu (0.54), and Mg (0.33), with higher concentrations in males.

#### Pollen

The first two principal axes of the PCA explained 50.98% of the variation (Fig. 3b). The pollen formed two groups. These groups were shifted along the 1st axis, explaining 28.01% of the variation. The elements contributing mostly to this shift were P (1st-axis loading: 0.81), S (0.76), Cu (0.68) and Zn (0.68), with higher concentrations in the pollen eaten by females.

#### Pupae

The first two principal components of the PCA explained 57.78% of the observed variation in elemental concentration (Fig. 3c). The pupae formed two groups along the 1st axis, explaining 39.43% of the variation. The elements contributing mostly to this shift were K (1st-axis loading: 0.86), Cu (0.85), Mn (0.75), P (0.73), Fe (0.71), and Zn (0.69), with higher concentrations in females, and Na (0.70), with higher concentrations in males.

#### Adults

A similar but not exactly the same shift as for pupae was observed for adults (Fig. 3d), with the 1st axis explaining 32.57% of the variation and primarily loaded by Ca (1st-axis loading: 0.85), K (0.79), Zn (0.79), Cu (0.79), P (0.62) and Na (0.50).

#### Cocoons

The first two principal components of the PCA explained 56.23% of the variation (Fig. 3e). The cocoons formed two groups along the 1st axis, explaining 32.57% of the variation. The elements contributing mostly to this shift were Zn (1st-axis loading: 0.95), Mn (0.86), Cu (0.82), and S (0.71), with higher concentrations in female cocoons, and Na (0.65), which was richer in male cocoons.

#### Excreta

The first two principal axes of the PCA (Fig. 3f) explained 53.23% of the observed variation. The excreta formed two groups along the 1st axis, explaining 32.57% of the variation. The elements contributing mostly to this shift were S (1st-axis loading: 0.94), Na (0.84), P (0.82), Mn (0.77) and K (0.68), with higher concentrations for females than for males.

### Allocation of elements to a bee and its cocoon

**Figure 4:**
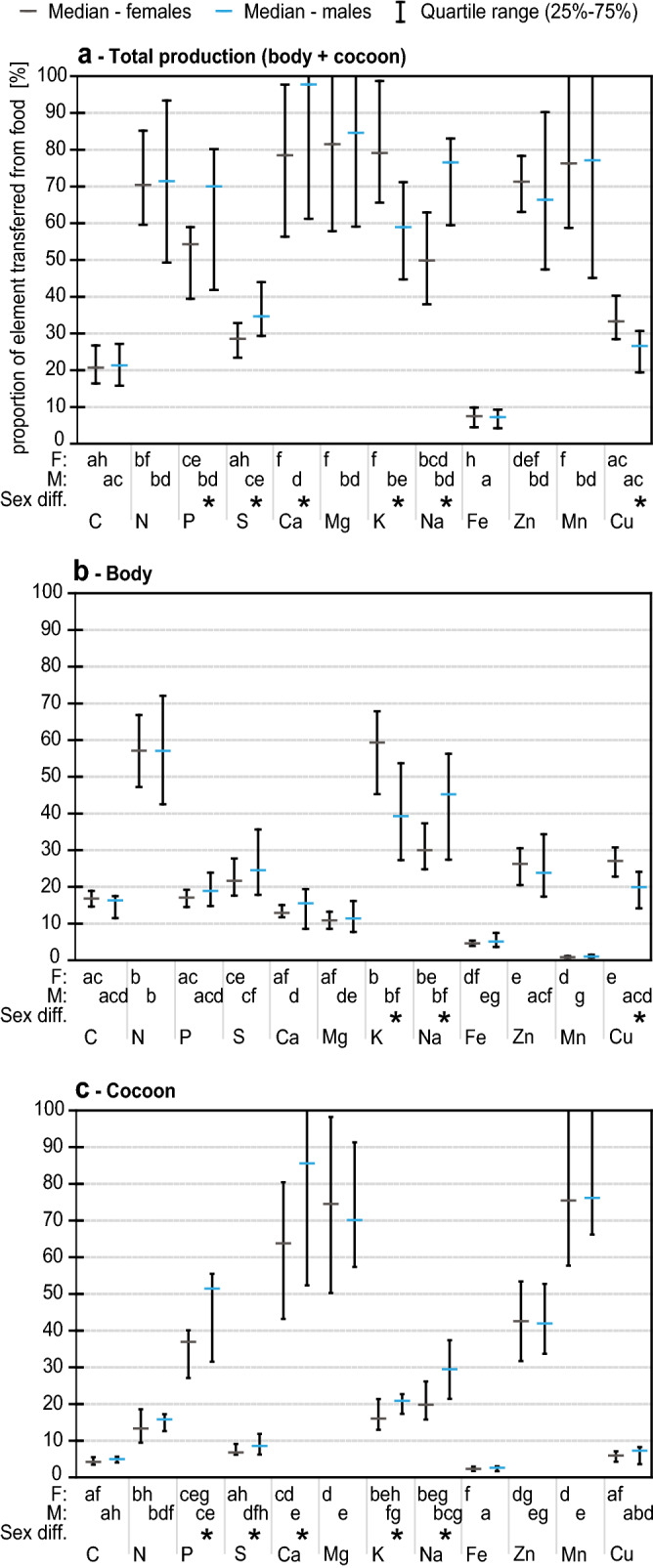
The transfer of matter from pollen to the body and cocoon differs between the sexes and elements. The Kruskal–Wallis test was used to assess the significance (p < 0.05) of differences between the values calculated for each element for a single sex, and the Mann–Whitney U test was used to assess the significance (p < 0.05) of differences between the values calculated for different sexes for each element. Sexual dimorphism is observed for P, S, Ca, K, Na and Cu.

Figure 4 shows the level of elemental transfer from pollen to adult body and its cocoon, calculated from data on the absolute masses of each element, with results of statistical tests analyzing differences between sexes and elements. Generally, atom transfer was dependent on element and sex. Considering total production (adult body and cocoon pooled together; Fig. 4a), the elements assimilated from pollen to the highest degree were Ca (median: females (F): 78.5%, males (M): 97.8%), Mg (median: both sexes (BS): 83.1%), Mn (BS: 76.3%), K (F: 79.1%, M: 59.0%), Na (F: 49.9%, M: 76.5%), N (BS: 70.4), P (F: 54.3%, M: 70.0%) and Zn (BS: 69.3%), and for most of these elements as well as for Cu, sexual differences in the level of elemental transfer were observed (Fig. 4a). Considering solely the adult body (Fig. 4a), the elements assimilated from pollen to the highest degree were K (F: 59.3%, M: 39.3%), N (BS: 57.1%), and Na (F: 30.0%, M: 45.2%). Considering solely the cocoon (Fig. 4a), the elements assimilated from pollen to the highest degree were Ca (F: 63.8%, M: 85.6%), Mn (BS: 76.0%), Mg (BS: 74.4%), P (F: 37.0%, M: 51.4) and Zn (BS: 42.0%).

**Figure 5:**
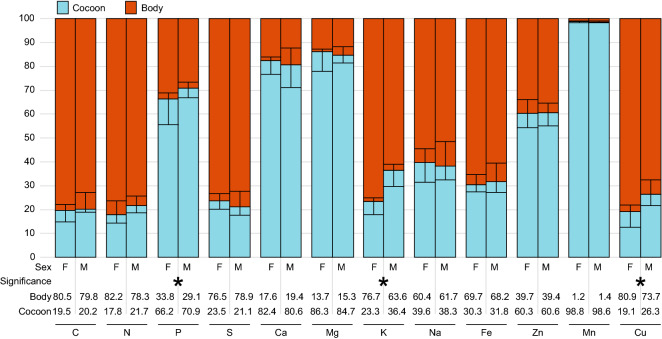
The allocation of elements assimilated from food differs between the sexes. Pattern of allocation of assimilated elements calculated for the total amount of elements composing the total production (body + cocoon): 100% = Mx_body_ + Mx_cocoon_. Mx = mass of element x. For every sex, N = 7. Statistical significance was calculated for each element separately (Mann–Whitney U, p < 0.05) and is indicated by asterisks. Median values are shown as lines dividing the body and cocoon, and whiskers show the minima and maxima. Median values for the percentage of an element allocated to the body/cocoon are given below the figure. Sexual dimorphism in the allocation of assimilated elements in the adult body and cocoon (fitness-enhancing excretion) is observed for K, P and Cu.

Figure 5 shows how the elements assimilated from pollen and not excreted were allocated between the adult body and the cocoon, as revealed by the analyses of sex differences in allocation pattern. Generally, this allocation was sex-dependent for P, K and Cu, with males allocating larger percentages of acquired atoms to cocoons and lower percentages to bodies (Fig. 5). For both sexes, the elements allocated mostly to the body (approx. 70–80%) were C, N, S and Cu, and the elements allocated mostly to the cocoon (70–100%) were Ca, Mg and Mn. Furthermore, almost 100% of the Mn acquired from food was allocated to the cocoon.

## Discussion

The most striking result of this study is how much phosphorus bees allocate to their adult body vs cocoon. Only approximately 25–45% of P assimilated from pollen was transferred to the body compared to 55–75% transferred to the cocoon embracing the body. This allocation pattern should be discussed in the context of predicted links between organismal growth, protein production and demand for P (growth rate hypothesis^2,29^). To meet increased demand for protein, developing and growing larvae have to maintain efficient translational machinery, including large amounts of RNA transcripts and rRNA that are rich in P. As a result, bee larvae have to assimilate large amounts of P to sustain their intense growth (in our study, bees assimilated approximately 54% of all P in pollen for females and 70% of all P in pollen for males). After turning into an adult, growth ceases, which should reduce the demand for P. In view of our results, more than half of P assimilated during larval development is further channeled to the cocoon at the stage of cocooning larva (in our case, approximately 66% of all P assimilated is allocated to the cocoon by females, and 71% of all P assimilated is allocated to the cocoon by males). Our finding shows that, at least for holometabolous insects, *C:N:P* stoichiometry and P concentrations in adult bodies, without considering cocoons, should not be used as a reliable proxy of the larval demand for P. What is thought-provoking is that when larval development is finished, surplus P is not defecated but is recycled for allocation into a cocoon. This suggests that in addition to fueling growth and development, P might have another fitness-related function—protection of a developing organism. Certainly, the effects of P on protecting the characteristics of cocoons await further research. We conclude that instead of relying solely on the stoichiometry of adult bodies, studies of holometabolous insects should refer to information on the chemical composition of the total production (adult body and its cocoon). This approach promises a more realistic picture of the links between the elemental phenotype, life history and fitness of adults and the nutritional constraints experienced by juvenile stages. Interestingly, our results showed that the assimilated P is differently allocated to adult bodies versus the cocoon in male and female bees, and we discuss this phenomenon later.

Various elements were transferred from pollen to the total production at different levels, indicating potential differences among elements in their limitations imposed on bee development. Given that the highest levels of the elemental transfer to the total production (here, arbitrarily set median transfer ≥ 70%; see  Fig. 4 and supplement) indicate a potential limitation by the food supply, our results suggest that limited access to the following elements hinders bee development in a sex-dependent manner (susceptibility of females (F) vs. males (M) to limitations indicated in brackets): Ca (F > M), Mg (F = M), K (only F), Na (only M), Mn (F = M), N (F = M) and P (only M). These results suggest that the commonly considered limitation by N and P^2^ does not play the most important limiting role in *O. bicornis*. For example, our results on pollen suggest that the risk of P limitation in female offspring can be reduced by the provisioning of P-rich pollen to larval cells by mother bees. These results validate the usefulness of the trophic stoichiometric ratio (TSR) model, which suggested similar conclusions based on purely theoretical consideration^23^.

In addition to P, the high allocation of Ca, Mg, Zn and Mn to the cocoon suggests that the scarcity of these elements in larval food might result primarily in the weakening of the protective functions of cocoons (compare with^30^ to see how evolutionary history and ecological pressures may shape allocation of specific metals to strengthen specific morphological structures). In contrast, N, S, K, Na, Fe and Cu were intensely transferred to the adult body, indicating that their scarcity may result in the underdevelopment of the bee body. Pupation of *O. bicornis* starts in nature in spring/early summer, approximately 45–50 days after egg laying to larval chambers. After another 50 days, an adult bee becomes formed inside the cocoon^31,32^. The adult spends the whole summer, autumn and winter inside its cocoon, emerging to the environment in the spring (next season), ready for reproduction (Fig. 1). This means that for a developing *O. bicornis*, a cocoon serves as protection for approximately 10 months, which suggests that its characteristics play a crucial role for bee fitness. Overall, our findings indicate that underdeveloped cocoons, which are sometimes observed in *O. bicornis*^33^, may indicate the scarcity of specific elements in food and/or a trade-off in the allocation of elements between the adult body and cocoon, which may have negative fitness implications.

To date, the majority of studies on nutrient cycling in terrestrial ecosystems highlighted the role of medium-sized or large mammalian herbivores in this process^34–37^, but similar effects of invertebrate herbivores are also probable. Indeed, we found considerable amounts of P, Ca, Mg, Zn and Mn built into cocoons, which, together with elements present in larval excreta, are left at nesting sites of bees. This locally enriched pool of elements can have a crucial impact on nutrient flows in ecosystems, ultimately shaping the stoichiometric niches of other community members. In the first step, these nutrients are mobilized from soil by plants and are deposited in pollen. Next, mother bees collect the nutrients with pollen, providing them to developing larvae. At this stage, the nutrient pool is rearranged stoichiometrically by a developing bee. Finally, nutrients are stored at the bee-nesting sites in the form of excreta and empty cocoons, which are stoichiometrically different from pollen. The biomass stored in nests have low values of *N:P* and *C:other elements* compared with those of pollen (especially cocoons having approx. *N:P* = 4 and *C:P* = 26; see molar ratios in supplement), which should affect decomposers, ultimately impacting the mineralization of elements and their availability for plants^36,38^.

We observed fundamental differences in the elemental phenotype between males and females of *O. bicornis*. Importantly, these differences occurred in multiple elements beyond those commonly studied (C, N and P) and were reflected in all components of the elemental budget, including the egg, adult body, pupal body, cocoon, food and excreta. These findings suggest that males and females are characterized by different acquisition, assimilation, allocation, and excretion of elements, which ultimately indicates that each sex has specific demands for nutrients, likely reflecting sex-differences in body structures and physiology. We interpret this phenomenon as phenotypical reflection of sex-specific selection pressures. Interestingly, our evidence shows an especially high utilization of P by females. What should be highlighted here is that the increased demand for P in female larvae was supported by “decisions” of mother bees regarding stoichiometric characteristics of larval provisions—the pollen deposited in female larval chambers was richer in P than that deposited in male chambers. Importantly, our comparison of P assimilation from pollen to the total production shows that the assimilation of P from pollen might be less costly for females since the proportion of P transferred from food is lower for females than for males. These data suggest that developing males have to perform heavier physiological work than females to assimilate all the needed P from pollen into their bodies and cocoons. Adult females are on average 1.7 times larger than adult males in terms of their dry mass, but even so, the P concentration is higher in adult female bodies than in male bodies (see supplement), contradictory to the expected P allometry^2,39^. One cause of this effect may be a higher concentration of DNA in polyploidic females than in haploidic males. An additional contributing factor might be the higher growth rate needed to reach the optimal size during larval development in females. In *O. bicornis*, body size is a key fitness component for females but not for males^40^. Specifically, large female size has significantly positive effects on fecundity, the size of the laid eggs, the investment in progeny and nest usurpation behavior, which in turn positively influence fitness in mason bees^41^. Finally, the higher P concentration in females may also be related to the investment of P-rich nucleic acids in growing oocytes to support early embryogenesis^42^. Females also assimilated Ca to a higher degree than males. Regarding Ca, cocoon calcification may be an adaptive modification allowing for the building of a strong cocoon while using a smaller amount of amino acids, and it was previously suggested that the origin of such mechanisms is caused by a trade-off between the deposition of N in the bee body and in the cocoon^28^. Within the context of results obtained here for P, we hypothesize that cocoon calcification may allow females to invest more P in adult bodies, supporting the abovementioned early embryogenesis^42^ and future reproduction. Interestingly, K and Na were also assimilated differently in both sexes. Males assimilated K to a higher degree and Na to a lower degree than females. We hypothesize that this phenomenon may be related to the role of Na in regulating the assimilation of N and P, especially considering that Na-P cotransporters directly influence bee physiology as well as the physiology of symbionts inhabiting bee guts and play a role in phosphate homeostasis and phosphate sensing at the cellular and organismal levels^34,43,44^. Since female bees have higher P demand than males, high Na assimilation and female-specific economy of Na and K might help meet this demand. Regarding S, which assimilated to a higher degree in females, and Cu, which assimilated to the higher degree in males, we are not aware of any physiological or morphological explanation for these sexual differences. Another interesting finding in terms of allocation is that the pool of P, K and Cu assimilated from food was differentially allocated to the adult body and cocoon, with females investing larger percentages of these elements into adult bodies and males investing larger percentages into cocoons. Since male and female cocoons differ in multi-elemental stoichiometry, they may have different mechanical and protective properties, which may be related to different locations of both sexes in the nest^6^; however, these issues have not been studied to date. Additional interesting sex differences in cocoon composition can be found in the concentrations of Zn and Mn, which are higher in female cocoons than in male cocoons (see supplement). Although much has been published regarding heavy metal toxicity (e.g.,^45–47^), heavy metals play essential roles in the functioning of organisms^48^. Zinc and Mn function to strengthen tissues and organs and increase the hardness and resistance of morphological structures^30^. The role of Zn and Mn in strengthening mandibles and organs related to reproduction and oviposition has only been briefly discussed in the literature^30^. Our study has shown that in the case of a solitary bee, the majority of assimilated Mn (almost 99%) and Zn (60%) is located in the cocoon. What is exceptionally interesting is that female cocoons have higher concentrations of these two elements than male cocoons (see supplement). This sexual dimorphism might be related to the higher production costs of female progeny and lower fitness value of single male progeny^31,40,41,49–51^. From the perspective of fitness maximization, the life strategy of a solitary bee might involve the production of female progeny characterized by increased protection from cocoons and male progeny characterized by increased strength of their sexual organs or mandibles.

The organismal stoichiometry, stoichiometric niche^15,52^ and nutritional limitation of individuals shape populations and communities of organisms via resource competition^2^. Unfortunately, intraspecific variability in elemental stoichiometry is not well documented, and most stoichiometrically explicit models treat populations as uniform pools of elements, although recent studies have attempted to address this issue^53–56^. This large-scale approach ignores the complexity of the population structure, thereby underestimating the variability among individuals and leading to an oversimplification of the conclusions about resource limitation. At the same time, the conflict between the sexes in reaching sex-specific nutritional optima or stoichiometric constraints imposed on a single life-history trait of a certain life stage/sex may drive trade-offs influencing fitness and thus negatively influence population growth^22,24,57^. Therefore, sexual dimorphism in nutritional demand and resource limitation may shape the population structure via the nutritional quality of available food. It is possible that each sex of *O. bicornis* has its own specific stoichiometric niche, reflecting the strategies of resource uptake and an optimization of nutrient use^52,58,59^*.*

Recently, the role of Na limitation in shaping ecological interactions was emphasized^34^; however, approximately 25 elements build molecules driving the functioning of every living organism on Earth^3^. The current study suggests that stoichiometric relations among all elements—not only C, N and P—may shape nutrient cycling (e.g., via relocation of stoichiometrically specific pollen and deposition of stoichiometrically specific detritus) and ecological interactions in ecosystems (e.g., bee-plant coevolution via bee needs for stoichiometrically balanced pollen); however, these relations are complicated via sexual dimension (described above) and via the interrelationship of elemental concentrations^60^.

Having access to balanced food resources is crucial for the development, health and fitness of bees^6,28^. Pollen quality varies widely across space and time and among plant species, which imposes a need for bees to select pollen that meets their nutritional demands^6,61,62^. There are large differences in floral preference between the sexes in bees^63^. This factor has not yet been considered in wild bee conservation efforts, and a better understanding of the nutritional ecology of wild bees is crucial for ensuring the effectiveness of conservation efforts. We still do not understand the basic mechanisms underlying the nutritional ecology of wild bees and the functioning of their populations. Bee conservation efforts are often based on simplistic assumptions regarding the nutritional ecology of one life stage (usually the adult) or sex (usually females)^6^. In reality, bee populations consist of individuals in various life stages and of different sexes. We believe that the new evidence presented here might be important for developing more effective management strategies for maintaining wild bee populations. This cannot be done without considering the complexity of relationships between the nutritional supply of pollen produced by different plants and the nutritional demands of whole bee populations as well as sex differences in the nutritional needs of bees at different life stages. An understanding of these links may be achieved via a synergistic approach linking physiological ecology and ecosystem ecology with the behavioral ecology and population ecology of bees. This approach will allow us to connect the basic physiological mechanisms underlying bee nutritional needs with the behavior (females collecting nutritionally specific pollen for daughters and sons) and functioning of bee populations (conflict in reaching sex- and life stage-specific nutritional optima) within the context of the ecosystem (the flora offering nutritionally balanced/unbalanced food). We propose ecological stoichiometry as an additional tool for achieving such synergy (see also^6,28^), one that is complementary to promising studies focusing on *protein-to-lipid* ratios^64^.

## Methods

### Study design

To obtain samples of different components of the elemental budget of *O. bicornis* (pollen, eggs, excreta, pupae, adults, cocoons), we used a trap nest, which consisted of 460 empty Phragmites stems (25–30 cm in length, 0.6–1 cm in diameter) embedded in a case (Fig. 1). The space inside stems offered a desirable habitat for mother bees to form larval cells. It was placed in Kraków, Poland (shrubbery/meadow, 50° 01′ 35″ N; 19° 54′ 05″ E; elevation: 213 m.a.s.l., MAAT: 8.7 °C, MAP: 679 mm). The nests were provisioned by mothers with pollen naturally available from plants in the area. The species composition of available flowering plants changes through the season; therefore, to ensure that uniform pollen loads were delivered to bees, only nests established over 1 days in May were studied. *O. bicornis* is a solitary bee; i.e., it neither lives in a hive nor forms a colony, and every nest is used by a different adult female. The complete description of the biology of the *Osmia* bee study system is given in the supplement.

At the egg laying stage, each larval cell that we studied contained one egg with a pollen load for the developing larva. At the pupal/adult stage, each cell contained a pupa/adult inside a cocoon, and excreta accumulated through larval development. Among 143 nests (stems; Fig. 1) completely filled with larval cells, we randomly sampled 90 nests to collect eggs and pollen, and 53 nests were left to allow bees to advance in development. We sampled 30 pupae from these nests and used the remaining 23 nests to collect adults, cocoons and excreta. To sex the specimens, we considered the first three cells at the entrance of a nest to be occupied by male offspring and the first three cells at the other end of the nest to contain female offspring (Fig. 1^6^). Only specimens from these cells were collected for our analyses. For the analysis of adults, pupae, cocoons, pollen and excreta, one specimen per analytical sample was used, and for eggs, we pooled 30 eggs of the same sex originating from 10 nests into single analytical samples. Consequently, our chemical information on adults, cocoons and excreta, but not on eggs and pollen, is derived from the same bee.

### Chemical analyses

Following our previous studies [e.g.,^5,21^] the C, N and S concentrations were determined using a Vario EL III automatic CHNS analyzer; the K, Ca, Mg, Fe, Zn, Mn, Cu and Na concentrations were determined using atomic absorption spectrometry (Perkin-Elmer AAnalyst 200 and Perkin-Elmer AAnalyst 800); and the P content was determined by colorimetry (MLE FIA). The samples were ground and homogenized using a mortar and coffee grinder and freeze dried to a dry mass. From each ground and homogenized sample, two analytical subsamples were obtained: (a) a liquid solution subsample suitable for analyzing the concentrations of K, Ca, Mg, Fe, Zn, Mn, Cu, Na and P and (b) a subsample with which to directly analyze the C, N and S concentrations^5,21^.

### Elemental budget

We used a series of analyses to explore the stoichiometry of pollen loads, eggs, pupae, adults, cocoons and excreta originating from male and female larval cells. In the first step, we analyzed the entire dataset (concentrations of all elements in different budget components) with the permutational analysis of variance (PERMANOVA) in PAST 3.26^65^, exploring whether the chemical composition differed between elemental budget components and sexes. This analysis was complemented by a principal component analysis (PCA) of the entire set of data performed in Canoco 5^66^, which helped us identify the elements that were the main drivers of patterns revealed by the PERMANOVA. In the next step, we focused specifically on stoichiometric differences between the sexes. For this purpose, we analyzed data separately for each component of the elemental budget. Each analysis involved a PCA on data on elemental concentrations for a given budget component, followed by a t-test (Statistica 13; p < 0.05) that compared scores of the most significant principal components between the sexes.

Additionally, we used a series of Mann–Whitney U tests (Statistica 13; p < 0.05) to compare raw data on the concentration of each element separately (12 elements) for males and females and for each component of the elemental budget (see the supplement). For comparative purposes, we also calculated the *C:N, C:P, N:P* and *C:other elements* atomic ratios separately for each sex and component of the elemental budget (see the supplement).

### Allocation of elements to a bee and its cocoon

The adult body and its cocoon represent the total production of a developing bee, entirely sustained from the pool of atoms available in the pollen supply^6,23^. We characterized the level of elemental transfer from food to total production by calculating what proportions of atoms of each element in food were deposited into an adult bee and its cocoon. Additionally, we explored what proportions of atoms of each element acquired from pollen were then allocated to a body of an adult bee versus its cocoon.

## Supplementary Information

Supplementary Information 1.

Supplementary Information 2.

## Data Availability

The authors declare that the data supporting the findings of this study are available within the paper (and its supplementary information files).
