# Supplementary material for: Stoichiometric niche, nutrient partitioning and resource allocation in a solitary bee are sex-specific and phosphorous is allocated mainly to the cocoon
Source: Sci Rep. 2021 Jan 12;11:652. doi: 10.1038/s41598-020-79647-7 (PMC7804283; doi:10.1038/s41598-020-79647-7)

Supplementary information for

How a bee is made: the sex-dependent elemental budget of the wild mason bee *Osmia bicornis* and its surprisingly intense phosphorous allocation into cocoons

Michał Filipiak, Michal Woyciechowski, Marcin Czarnoleski

S1 Appendix. Supplementary results on the concentration of each element for males and females and for each component of the elemental budget

Eggs

The concentrations of C and N in dry mass did not differ between the sexes for eggs (Supplementary Fig. S1). The median concentrations and molar ratios pooled between the two sexes were: C – 47.69%, N – 7.44%, *C:N* – 7.32. The percentage of P in dry mass was sex-dependent. The median concentrations and molar ratios were: female P – 0.64%, *C:P* – 195.3, *N:P* – 26.9; male P – 0.89%, *C:P* – 141.9, *N:P* – 17.7. Statistically significant differences between the sexes were also observed for the S concentration (median: females – 0.33%, males – 0.25%) and for the Cu concentration (median: females – 10.78 ppm, males – 20.43 ppm; Supplementary Fig. S1).

Pollen

The relative C and N contents in dry mass did not differ between sexes for the pollen eaten during larval development (Supplementary Fig. S1). The median contents and molar ratios pooled between the two sexes were C – 46.67%, N – 3.92%, and atomic *C:N* – 13.6. The percentage of P in dry mass was sex-dependent. The median contents and molar ratios were as follows: female P – 0.60%, *C:P* – 199.3, *N:P* – 13.3; male P – 0.47%, *C:P* – 254.1, *N:P* – 19.8 (Supplementary Fig. S1). Statistically significant differences between sexes were also found for the S (median: females – 0.15%, males – 0.13%) and Cu (median: females – 10.78 ppm, males – 20.43 ppm) concentrations in eggs (Supplementary Fig. S1).

Pupae, adults and cocoons

The relative C and N contents in dry mass did not differ between the sexes for pupae, adults or cocoons (Supplementary Fig. S1). The median contents and molar ratios pooled between the two sexes were: pupal C – 54.00%, N – 10.43%, *C:N* – 6.0; adult C – 48.43%, N – 14.11%, *C:N* – 4.0;

cocoon C – 38.80%, N – 10.74%, *C:N* – 4.2. The percentage of P in dry mass was sex-dependent for pupae and adults, but not for cocoons. The median contents and molar ratios were: pupal female P – 0.42%, *C:P* – 324.5, *N:P* – 56.2; pupal male P – 0.38%, *C:P* – 367.6, *N:P* – 59.2; adult female P – 0.60%, *C:P* – 203.8, *N:P* – 53.5; adult male P – 0.55%, *C:P* – 254.1, *N:P* – 19.8; male + female cocoon P – 3.82, *C:P* – 27.6, *N:P* – 6.7 (Supplementary Fig. S1). Statistically significant differences in elemental concentrations between the sexes were also observed for S in cocoons (median: females – 0.21%, males – 0.18%), Ca in adults (females – 0.09%, males – 0.10%), K in pupae (females – 1.58%, males – 1.28%), K in adults (females – 2.18%, males – 1.61%), Na in adults (females – 210.74 ppm, males – 239.81 ppm), Na in cocoons (females – 451.02 ppm, males – 500.60 ppm), Fe in pupae (females – 71.08 ppm, males – 62.02 ppm), Zn in pupae (females – 83.14 ppm, males – 64.18 ppm), Zn in cocoons (females – 538.53 ppm, males – 386.21 ppm), Mn in cocoons (females – 1090.20 ppm, males – 828.80 ppm), Cu in pupae (females – 16.58 ppm, males – 10.96 ppm), Cu in adults (females – 21.42 ppm, males – 14.56 ppm) and Cu in cocoons (females – 16.84 ppm, males – 15.44 ppm) (Supplementary Fig. S1).

Excreta (feces)

The relative C content in excreta did not differ between the sexes, with a total of 51.00% C (median of values pooled between the sexes; Supplementary Fig. S1). The percentages of N and P were sex-dependent. The median contents and molar ratios were: female N – 4.92%, P – 1.33%, *C:N* – 12.4, *C:P* – 98.7, *N:P* – 8.4; male N – 3.72%, P – 0.73%, *C:N* – 13.6, *C:P* – 181.7, *N:P* – 12.0. Statistically significant differences between the sexes were also found for S (median: females – 0.48%, males – 0.36%), Na (females – 243.69 ppm, males – 179.92 ppm), Fe (females – 1349.95 ppm, males – 1087.50 ppm) and Mn (females – 65.74 ppm, males – 41.66 ppm).

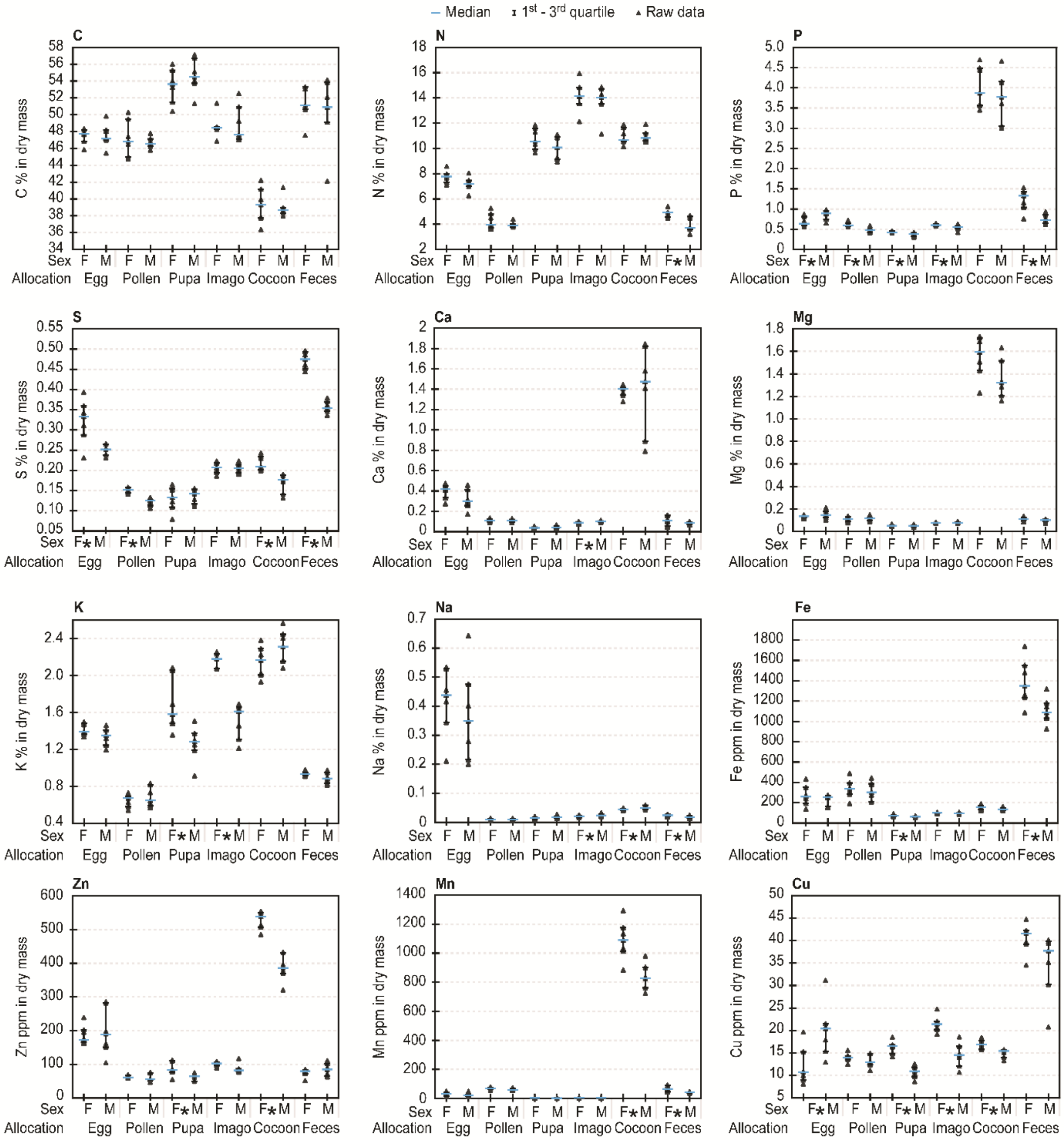

Supplementary Fig. S1. For both sexes, various components of elemental budgets are composed of different concentrations of elements. Multi-elemental compositions (relative contents in dry mass) of *O. bicornis* eggs, pupal bodies, adult bodies, cocoons, excreta, i.e., feces excreted during larval development, and pollen eaten during larval development. For each sample type (egg, pupa, adult, cocoon, excreta, pollen), sexual differences were tested separately for the concentrations of every element (Mann-Whitney U, $p<0.05$, $N=7$). Statistically significant differences are denoted with an asterisk. Sex differences are observed in the concentrations of P, S, Ca, K, Na, Fe, Zn, Mn and Cu but not in the concentrations of C, N and Mg.

S2 Appendix. Osmia solitary bee study system

The unique biology of solitary bees enables the exact food eaten and 100% of the feces excreted during development to be collected precisely for a single specimen under natural conditions, and it allows for the easy preparation of laboratory feeding experiments. This capability makes solitary bees a perfect model organism for studies of ecological stoichiometry.

The female lays its eggs in cracks or holes, such as in fractured wood and rocks or cracks in building walls, preferring empty Phragmite stems. The nests are fine-lined, and they consist of a few to several dozen compartments (larval cells) that are closed on both sides. One egg is laid in each cell after a defined amount of pollen is deposited, and the female determines the sex of the egg by either fertilizing it or not. The pollen load that the mother provides for her progeny is larger for female than for male offspring, and the cells prepared for females are provisioned first, and are, thus, located in the rear of the nest, which allows easy sex identification at any developmental stage. The pollen load is subsequently utilized by the larva during growth and development. Larvae pupate in the summer, and bees overwinter in their cocoons as adults and emerge the following spring. All the excreta produced during the larval period are stored in a cell together with the cocoon and can be easily collected. Therefore, the whole elemental budget can be easily studied for a single specimen, from an egg through the pollen eaten by a single larva to an adult specimen that built itself and its cocoon based on this pollen. All the excreta produced during the entire larval period may be also easily included in such a study. Adult females are always larger and look different than males. In case of females (and not for males), size is correlated with fitness, as the size has significantly positive effects on the female fecundity, size of laid eggs, the investment in progeny and nest usurpation behavior.

In our work, we used *Osmia bicornis* L. – a generalist wild bee inhabiting almost all of Europe, the northern part of Africa and western Asia. The study system may also be used outside of Europe based on native solitary bee species; for example, *Osmia lignaria* Say is a model solitary bee species in North America. Although more than 300 species of *Osmia* exist in the world, they are almost exclusively limited to the northern hemisphere.

<u>Life cycle in brief:</u>

- In early spring, when the air temperature is high enough, adults emerge from cocoons and come out of their nests.
- After 1-5 (2 on average) days, females start building new nests in which they form larval cells, filled with pollen and eggs (one cell always contain one pollen load and a single egg, and daughters are provided with higher amounts of pollen than sons). The mother bee decides on the

egg sex (female eggs are laid first, in the rear side of the nest, followed by male eggs, in the front side). The sex ratio of the whole brood is usually slightly increased towards males.

- Larvae hatch after 3-10 days and spend 15-35 days as actively feeding larvae, followed by the spinning larvae stage, lasting 3-10 days, prepupa (15-40 days) and pupa (10-50 days). Mortality at this stage of development (from egg to pupa) is approximately 10-30%.
- After pupating, adult specimens inside their cocoons prewinter from summer to autumn and spend the whole winter and the beginning of the spring inside the cocoons, until the air temperature reaches approximately a dozen or so degrees centigrade. Mortality at this stage (from pupated adult to adult leaving its cocoon) is approximately 0-10%.

A detailed description of Osmia mason bee nesting biology may be found here:

Bosch, J., Sgolastra, F., & Kemp, W. P. (2008). Life Cycle Ecophysiology of Osmia Mason Bees Used as Crop Pollinators. In *Bee Pollination in Agricultural Eco-systems* (Vol. 1, pp. 83–105). doi: 10.1093/acprof:oso/9780195316957.003.0006; https://www.oxfordscholarship.com/view/10.1093/acprof:oso/9780195316957.001.0001/acprof-9780195316957-chapter-6

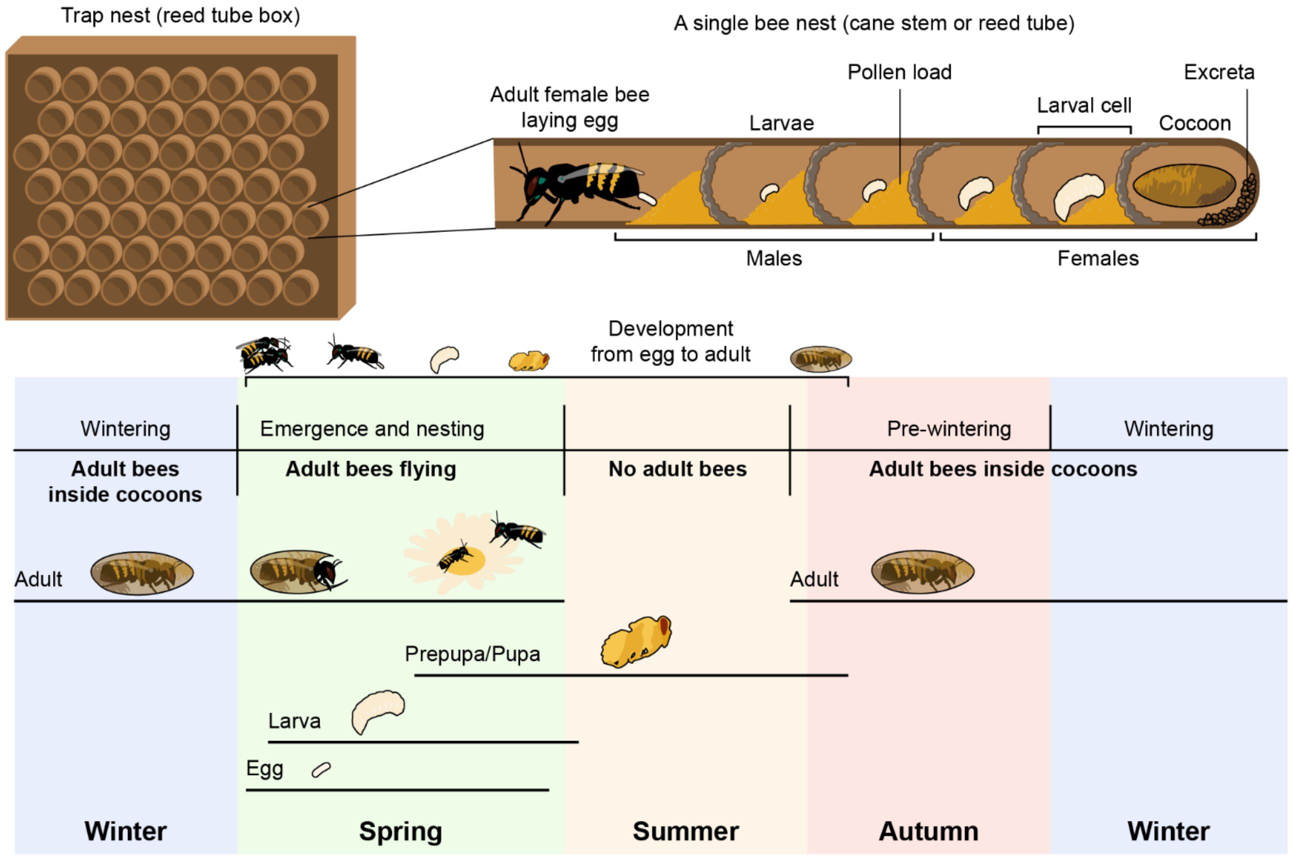

Supplement: Supplementary file 1 — Supplementary Information 1. [file 41598_2020_79647_MOESM1_ESM.pdf]
